# Chemical Composition, Enantiomeric Distribution, and Antifungal Activity of the Oleoresin Essential Oil of *Protium amazonicum* from Ecuador

**DOI:** 10.3390/medicines4040070

**Published:** 2017-09-23

**Authors:** Prabodh Satyal, Chelsea N. Powers, Rafael Parducci V., Robert L. McFeeters, William N. Setzer

**Affiliations:** 1 Department of Chemistry, University of Alabama in Huntsville, Huntsville, AL 35899, USA; prabodhsatyal@gmail.com (P.S.); cnp0007@uah.edu (C.N.P.); robert.mcfeeters@uah.edu (R.L.M.); 2 Aromatic Plant Research Center, 615 St. George Square Court, Suite 300, Winston-Salem, NC 27103, USA; 3 Saintoil S.A., Magnolias 57, Quito 17-22-20108, Pinchachas, Ecuador; Saintoil@aol.com

**Keywords:** essential oil composition, *Protium amazonicum*, Burseraceae, copal, breu, δ-3-carene, chiral gas chromatography, antifungal activity

## Abstract

**Background:** *Protium* species (Burseraceae) have been used in the treatment of various diseases and conditions such as ulcers and wounds. **Methods:** The essential oil from the oleoresin of *Protium amazonicum* was obtained by hydrodistillation and analyzed by GC-MS, GC-FID, and chiral GC-MS. *P. amazonicum* oleoresin oil was screened for antifungal activity against *Candida albicans*, *Aspergillus niger*, and *Cryptococcus neoformans*. **Results:** A total of 54 components representing 99.6% of the composition were identified in the oil. The essential oil was dominated by δ-3-carene (47.9%) with lesser quantities of other monoterpenoids α-pinene (4.0%), *p*-cymene (4.1%), limonene (5.1%), α-terpineol (5.5%) and *p*-cymen-8-ol (4.8%). Chiral GC-MS revealed most of the monoterpenoids to have a majority of *levo* enantiomers present with the exceptions of limonene and α-terpineol, which showed a *dextro* majority. *P. amazonicum* oleoresin oil showed promising activity against *Cryptococcus neoformans*, with MIC = 156 μg/mL. **Conclusions:** This account is the first reporting of both the chemical composition and enantiomeric distribution of the oleoresin essential oil of *P. amazonicum* from Ecuador. The oil was dominated by (−)-δ-3-carene, and this compound, along with other monoterpenoids, likely accounts for the observed antifungal activity of the oil.

## 1. Introduction

*Protium amazonicum* (Cuatrec.) Daly belongs to the Burseraceae, which is comprised of 640 species representing 18 genera throughout the world, mainly distributed in the Neotropics and North Africa [1]. The main characteristic of the Burseraceae is the exuding aromatic resin [2,3], which is known as “copal” in Spanish [4] and “breu” in Portuguese [5]. *Protium* spp. have been used in the treatment of various diseases and conditions such as ulcers and wounds, to treat headaches, toothaches, and rheumatism [2], because of their anti-inflammatory [6,7], antinociceptive [8,9], antineoplastic [10], and gastroprotective [11,12] properties. The Yanomami people of Brazil use the resin of *P. fimbriatum* to treat respiratory infections [13]. *Protium* oleoresins have been characterized in terms of color, age, odor, as well as volatile and non-volatile chemical characteristics (Table 1) [5,14]. Because of the importance of *Protium* oleoresins in traditional medicine and because no previous work had been carried out on *P. amazonicum*, we wished to chemically characterize the oleoresin essential oil of *P. amazonicum*; this information should add to our understanding of *Protium* oleoresin chemistry.

## 2. Materials and Methods

### 2.1. Essential Oil

The oleoresin (relatively fresh, yellow, with a terpenic odor) of *P. amazonicum* was collected from Quito, Ecuador (0°14′0″ S, 78°31′0″ W, 3000 m above sea level). The tree was identified by Rafael Parducci, and a voucher specimen has been deposited in Saintoil S.A. The essential oil was obtained by hydrodistillation using a Clevenger apparatus as previously described [29] to give the essential oil.

### 2.2. Gas Chromatography-Mass Spectrometry (GC-MS)

The oleoresin essential oil of *P. amazonicum* was analyzed by GC-MS using a Shimadzu GC-MS-QP2010 Ultra (Shimadzu Corp., Columbia, MD, USA) operated in the electron impact (EI) mode (electron energy = 70 eV), with a scan range of 40–400 atomic mass units (amu), a scan rate of 3.0 scans/s, and the GC-MS Solution software (Shimadzu GC-MS-QP2010 Ultra, Columbia, MD, USA). The GC column was ZB-5MS fused silica capillary column (Phenomenex Inc., Torrance, CA, USA) (30 mL × 0.25 mm ID) with a (5% phenyl)-polymethylsiloxane stationary phase with a film thickness of 0.25 μm. The carrier gas was helium with a column head pressure of 551.6 kPa and flow rate of 1.37 mL/min. The injector temperature was 250 °C, and the ion source temperature was 200 °C. The GC oven temperature program was programmed for 50 °C initial temperature, the temperature increased at a rate of 2 °C/min to 260 °C. A 5% *w*/*v* solution of the sample in CH_2_Cl_2_ was prepared and 0.1 µL was injected with a splitting mode (30:1). Identification of the oil components was based on their retention indices determined by reference to a homologous series of *n*-alkanes, and by comparison of their mass spectral fragmentation patterns with those reported in the literature [30], and stored in the MS library.

### 2.3. Gas Chromatography—Flame Ionization Detection

The gas chromatograph was a Shimadzu GC 2010 (Shimadzu Corp., Columbia, MD, USA) equipped with a flame ionization detector, a split/splitless injector, and autosampler AOC-20i (Shimadzu Corp., Columbia, MD, USA). The capillary column was a ZB-5MS (Phenomenex Inc., Torrance, CA, USA) with a film thickness of 0.25 μm. The column temperature was programmed, 50–250 °C at 2 °C/min, the injector temperature was 250 °C, the detector temperature was 280 °C, the carrier gas was nitrogen, and the flow rate was maintained at 1.0 mL/min. Injection mode split with a split ratio of 1:100. The injected volume was 0.3 µL of diluted oil (1:10 *v*/*v* with CH_2_Cl_2_). The percent composition of the oleoresin essential oil was calculated from raw peak areas without standardization.

### 2.4. Chiral Gas Chromatography—Mass Spectrometry

Chiral analysis of the *P. amazonicum* oil was performed on a Shimadzu GCMS-QP2010S (Shimadzu Corp., Columbia, MD, USA) operated in the EI mode (electron energy = 70 eV), scan range = 40–400 amu, scan rate = 3.0 scans/s. GC equipped with a Restek B-Dex 325 capillary column (30 m × 0.25 mm ID × 0.25 μm film) (Restek Corp., Bellefonte, PA, USA). Oven temperature was started at 50 °C, and then gradually raised to 120 °C at 1.5 °C/min. The oven was then raised to 200 °C at 2 °C/min and held for 5 min. Helium was the carrier gas and the flow rate was maintained at 1.8 mL/min. The sample was diluted 3% *w*/*v* with CH_2_Cl_2_ and then a 0.1 µL sample was injected in a split mode with a split ratio of 1:45. The enantiomers of each monoterpene were identified by comparison of retention times to authentic samples obtained from Sigma-Aldrich (Milwaukee, WI, USA).

### 2.5. Antifungal Screening

The broth microdilution method was performed to determine antifungal activity as previously reported [31,32]. Briefly, cultures of *Candida albicans* (ATCC 18804) and *Cryptococcus neoformans* var. *neoformans* (ATCC 24067) were initially grown on potato dextrose agar (PDA) plates for 72 h at 37 °C. A single colony was used to inoculate approximately 5 mL of potato dextrose broth (PDB) which was subsequently grown for an additional 24 h at 37 °C. *Aspergillus niger* (ATCC 16888) cultures were grown on PDA plates for 5 days at room temperature (RT, 22 °C). *A. niger* conidia were collected, placed in PDB, and filtered through sterile cheesecloth into fresh PDB. The absorbance of the fresh solution was read at 625 nm and adjusted accordingly with PDB to an absorbance of 0.15. Minimum inhibitory concentrations (MICs) were determined in triplicate using 96-well plates. *C. albicans* and *C. neoformans* were diluted in 3-(*N*-morpholino)propanesulfonic acid (MOPS) buffered Roswell Park Memorial Institute (RPMI) medium to 2000 cells/mL whereas *A. niger* was diluted with PDB to an OD_625_ of 0.15. Initially, 50 μL of MOPS buffered RPMI was added to each well of the plate. In the first row, 50 μL of essential oil was added and mixed well, then 50 μL of this mixture was removed and then added to the medium in the next row. This serial dilution process was repeated for each row of the plate, with the removed volume from the last row being discarded. To each well was added 50 μL of cells to achieve a final volume of 100 μL. *C. albicans* and *C. neoformans* were incubated at 37 °C for 48 h. The *A. niger* plates were incubated at RT for 6 days. The MIC was determined from turbidity or growth on the plates in comparison to positive and negative controls. In order to verify the results, MIC determinations were carried out in nine replicates. A combination of Cyprodinil and Fludioxonil served as the positive control with MOPS buffered RPMI serving as the negative control.

## 3. Results and Discussion

### 3.1. Chemical Composition

**Figure 1:**
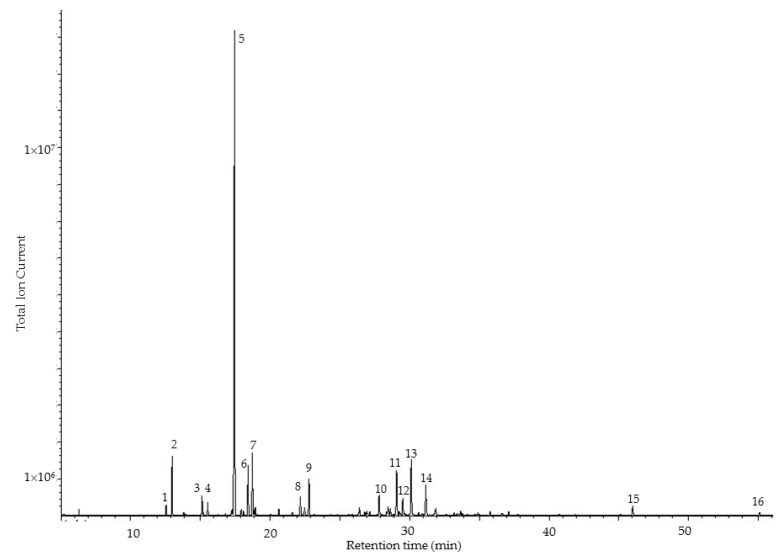
Gas chromatogram of the oleoresin essential oil of *Protium amazonicum* from Ecuador. 1, α-thujene; 2, α-pinene; 3, 3,7,7-trimethyl-1,3,5-cycloheptatriene; 4, β-pinene; 5, δ-3-carene; 6, *p*-cymene; 7, limonene; 8, *m*-cymenene; 9, *p*-cymenene; 10, α-phellandren-8-ol; 11, *m*-cymen-8-ol; 12, *p*-cymen-8-ol; 13, α-terpineol; 14, 4-methyleneisophorone; 15, α-*trans*-bergamotene; 16, caryophyllene oxide.

The clear pale yellow oleoresin essential oil from *P. amazonicum* was obtained in 0.3% yield and analyzed by GC-MS and GC-FID. From a total of 56 peaks, 99.6% of the compounds were identified in the oil (Table 2). The major components of the resin oil were identified as δ-3-carene (47.9%), α-pinene (4.0%), *p*-cymene (4.1%), limonene (5.1%), α-terpineol (5.5%) and *p*-cymen-8-ol (4.8%) (see Figure 1). δ-3-Carene has been reported as a major component in several *Protium* spp. oleoresin essential oils, including *P. decandrum* and *P. heptaphyllum* [15]; however, in most oleoresin essential oils from *Protium*, δ-3-carene is a minor component or unobserved (see Table 1). *Protium* oleoresin oils show wide variation in chemical composition, depending on species as well as age and color of the resin (Table 1). The age of an oleoresin has a distinct effect on the chemical composition. Some monoterpenes have been found to undergo oxidation upon exposure to atmospheric oxygen [33,34,35], including oleoresin monoterpenoids [20]. In addition, fresh oleoresin from the same species shows wide variation in chemical composition. Thus, for example, the essential oil from fresh oleoresin of *P. heptaphyllum* collected from the Restinga of Carapebus, Rio de Janeiro state, Brazil, had myrcene (35.0%) and α-pinene (27.0%) as the major components [10]; the fresh resin oil from Reserva da Campina, Amazonas, Brazil, was rich in *p*-cymene (36.0%), α-terpinene (18.0%), and γ-terpinene (12.0%) [23]; and the fresh resin oil from Crato, Ceara, Brazil was dominated by terpinolene (28.5%), α-phellandrene (16.7%), and limonene (16.9%) [24]. The oleoresin in this present work is a relatively fresh resin, reflected in the high concentration of δ-3-carene.

### 3.2. Enantiomeric Distribution

**Figure 2:**
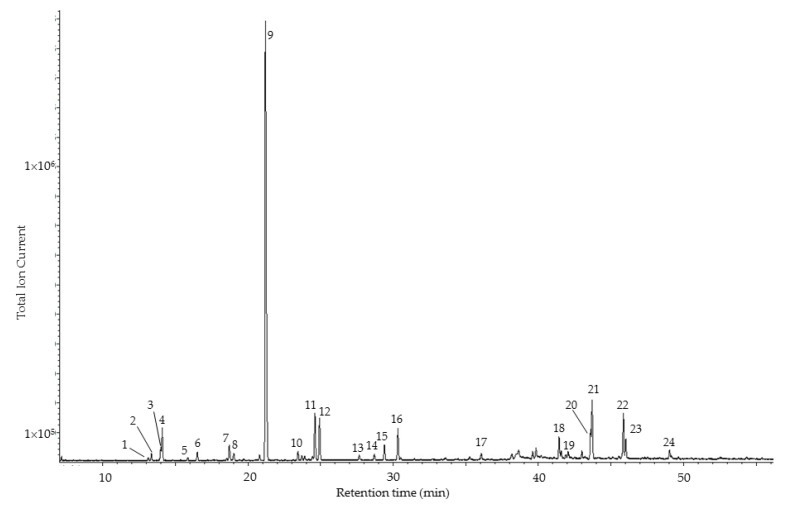
Chiral gas chromatogram of the oleoresin essential oil of *Protium amazonicum*. 1, (+)-α-thujene; 2, (−)-α-thujene; 3, (+)-α-pinene; 4, (−)-α-pinene; 5, (+)-β-pinene; 6, (−)-β-pinene; 7, 3,7,7-trimethyl-1,3,5-cycloheptatriene; 8, 1,8-cineole; 9, (−)-δ-3-carene; 10, (−)-limonene; 11, (+)-limonene; 12, *p*-cymene; 13, α-terpinolene; 14, γ-terpinene; 15, *m*-cymenene; 16, *p*-cymenene; 17, camphor; 18, α-phellandren-8-ol; 19, (−)-α-terpineol; 20, eucarvone; 21, (+)-α-terpineol; 22, *m*-cymen-8-ol; 23, *p*-cymen-8-ol; 24, α-*trans*-bergamotene.

Chiral GC-MS analysis was performed to evaluate the enantiomeric distribution of the monoterpenes present in *P. amazonicum* essential oil (see Table 3 and Figure 2). The levorotatory (−)-enantiomer of δ-3-carene was found to be the exclusive stereoisomer while the (−)-enantiomers of α-pinene, β-pinene, and sabinene predominated over the (+)-isomers. The (+)-enantiomers of limonene and α-terpineol, on the other hand, were dominant. The hexane root extract of *Angelica archangelica* showed predominantly (+)-δ-3-carene, but the (−)-enantiomer was detected [36]. Only the (+)-enantiomer of δ-3-carene was detected in *Pinus sylvestris* (Pinaceae) essential oils, while (−)-limonene predominated [37]. The (+)-enantiomer of limonene is the more common, especially in *Citrus* (Rutaceae) essential oils [38,39,40,41,42]. *Micromeria fruticosa* (Lamiaceae) essential oil showed exclusively (+)-α-terpineol while (−)-α-terpineol was found in *Laurus nobilis* (Lauraceae) essential oil [43]. Analysis of the essential oil from the unripe fruits of *Pistacia vera* showed a predominance of (+)-α-pinene, (+)-limonene, (+)-β-pinene, and exclusively (−)-α-terpineol [44]. Although δ-3-carene was relatively abundant in this oil (2.7%), the enantiomeric distribution was unfortunately not reported. The oleoresin of *Boswellia carterii* (Burseraceae) from Ethiopia was composed of (+)-α-thujene, (−)-α-pinene, and (−)-limonene, but the enantiomeric distribution of δ-3-carene was not determined [45]. In contrast, *B. carterii* resin oil from Somalia showed (−)-α-thujene, (−)-α-pinene, and (−)-limonene predominating, while *B. sacra* resin oil from Oman had (+)-α-thujene, (+)-α-pinene, (+)-β-pinene, and (−)-limonene predominating [46].

### 3.3. Antifungal Activity

The oleoresin essential oil of *P. amazonicum* demonstrated antifungal activity against *C. albicans*, *C. neoformans*, and *A. niger*. *C. neoformans* was most potently inhibited with a promising MIC of 156 μg/mL. Inhibition of *C. albicans* (MIC = 313 μg/mL) was also rather promising whereas inhibition of *A. niger* was relatively weak (MIC = 1250 μg/mL). The major component in *P. amazonicum* oil, δ-3-carene, has shown antifungal activity against several fungi, including *C. albicans* [47]. In addition, minor monoterpenoid components in the oil, α-pinene [48], limonene [49], and α-terpineol [50], have also shown antifungal activities. 

The antifungal mechanisms of activity of monoterpenoids are poorly understood. It has been suggested that these hydrophobic compounds disrupt the cytoplasmic membranes or membrane proteins of fungal cells, leading to cytoplasmic leakage, cell lysis, and death [51]. Chirality of monoterpenoids, therefore, may not play a critical role in antimicrobial activity. Nevertheless, Kusumoto and co-workers have shown that (+)-α-pinene showed significantly better antifungal activity against *Heterobasidion parviporum* than (−)-α-pinene [52]. Likewise, Filipowicz et al. showed (−)-β-pinene to be slightly more active than (+)-β-pinene against *Candida albicans* [53], and Omran and co-workers found that (−)-limonene had better antifungal activity than (+)-limonene [54]. (+)-δ-3-Carene has shown antifungal activity against several fungal strains [47], but there are apparently no reports on antifungal activity of (−)-δ-3-carene, which is not commercially available. Overall, these findings indicate that *P. amazonicum* resin oil has promising potential for further antifungal consideration, in particular against *C. neoformans* and potentially other yeast-like fungi.

## 4. Conclusions

This is the first reported chemical analysis of the oleoresin essential oil of *Protium amazonicum*. The *P. amazonicum* resin oil collected in Ecuador was dominated by (−)-δ-3-carene and is therefore, an excellent source of this enantiomer. The abundance of this compound, along with other monoterpenoids, likely account for the observed antifungal activity of the oil. The activity against *Cryptococcus neoformans* and *Candida albicans* indicates promise against these opportunistic fungal pathogens. Additional research into this tree species and other *Protium* species, their chemistry and their biological activities, is needed.

## Figures and Tables

**Table 1:** A brief review of *Protium* oleoresin traditional medicinal uses, biological properties, and essential oil compositions. ^a^

Species	Traditional Medicinal Uses and/or Biological Activities	Major Components	Ref.
*P. altsonii* (sucuruba)		*p*-cymene (16.3%), γ-cadinene (9.5%), γ-gurjunene (5.2%)	[15]
*P. bahianum*	Treatment of wounds, ulcers, inflammation, and as an insect repellent	Fresh resin: *p*-cymene (18.3%), α-phellandrene (14.0%), tricyclene (11.4%), β-phellandrene (9.1%), β-pinene (6.6%)	[16]
*P. bahianum*	Acaricidal activity (*Tetranychus urticae*)	Aged resin: (*E*)-β-santalol acetate (83.1%)	[16]
*P. decandrum*		α-trans-bergamotene (47.7%), α-cis-bergamotene (6.5%), β-caryophyllene (5.9%), *ar*-curcumene (5.2%)	[17]
*P. decandrum* (black breu)		δ-3-carene + *iso*-sylvestrene (40.9%), *p*-cymene (13.4%), limonene + β-phellandrene (20.3%)	[15]
*P. decandrum* (white breu)	Burning and inhaling smoke to treat headache	*p*-cymene (32.4%), α-phellandrene (21.0%), α-pinene (19.0%), limonene + β-phellandrene (12.0%)	[15]
*P. heptaphyllum*	Antimicrobial (*Candida albicans*, MIC = 1.25 μg/mL; *Staphylococcus aureus*, MIC = 2.5 μg/mL)	α-pinene (10.5%), α-phellandrene (16.7%), *p*-cymene (6.0%), limonene (16.9%), terpinolene (28.5%)	[18]
*P. heptaphyllum*	Antinociceptive (mouse model)	1,8-cineole (58.7%), α-terpinene (13.7%), α-phellandrene (10.4%), γ-terpineol (7.7%)	[9]
*P. heptaphyllum*	Anti-inflammatory (rat model)	limonene (50.0%), (*E*)-β-ocimene (11.8%), 1,8-cineole (10.9%), *p*-cymene (10.8%), α-phellandrene (10.0%)	[7]
*P. heptaphyllum*	Anti-genotoxic activity	terpinolene (32.7–37.8%), *p*-cymene (7.9–38.1%), limonene (0–2%), δ-3-carene (0–15.0%), α-thujene (0–1.1%), *p*-cymen-8-ol (2.5–10.1%)	[19]
*P. heptaphyllum*		Fresh resin: terpinolene (28.2–69.7%), *p*-cymene (4.3–23.3%), α-pinene (3.6–14.6%), α-terpinene (3.1–10.4%), limonene (6.4–10.1%), *p*-cymen-8-ol (2.7–9.8%)	[20]
*P. heptaphyllum*		Aged resin: *p*-cymene (18.7–43.0%), terpinolene (8.8–21.6%), α-pinene (3.5–17.8%), α-limonene (5.8–1.6%), *p*-cymen-8-ol (8.2–31.8%)	[20]
*P. heptaphyllum*		Fresh resin: myrcene (35.0%), α-pinene (27.0%), sabinene (11.0%), β-caryophyllene (7.2%)	[10]
*P. heptaphyllum*	Cytotoxic on SP2/0 (murine plasmocytoma) and J774 (murine monocytic macrophage) cell lines	Freshly tapped resin: terpinolene (28.0%), *p*-cymene (16.0%), α-pinene (8.7%), α-terpinene (6.6%), limonene (5.5%), *p*-cymen-8-ol (5.6%)	[10]
*P. heptaphyllum*	Antibacterial (*Streptococcus mutans*, MIC 0.13 μg/mL)	tricyclene (11.1%), *p*-cymene (26.7%), terpinolene (35.8%), *p*-cymen-8-ol (10.1%)	[21]
*P. heptaphyllum*	Vasorelaxant (rat upper mesenteric artery ring, IC_50_ 316 μg/mL)	δ-3-carene (5.1%), *p*-cymene (17.0%), limonene (34.5%), 1,8-cineole (20.6%), α-terpineol (9.8%)	[22]
*P. heptaphyllum*		α-phellandrene (7.0%), *p*-cymene (26.9%), limonene (28.9%), α-terpineol (18.4%)	[22]
*P. heptaphyllum*		Fresh resin: α-terpinene (18.0%), *p*-cymene (36.0%), γ-terpinene (12.0%)	[23]
*P. heptaphyllum*		Aged resin: *p*-cymene (11.0%), terpinolene (15.0%), *p*-cymenene (5.3%), *p*-cymen-8-ol (11.0%), dillapiole (16.0%)	[23]
*P. heptaphyllum*		Fresh resin: α-pinene (10.5%), α-phellandrene (16.7%), *p*-cymene (6.0%), limonene (16.9%), terpinolene (28.5%)	[24]
*P. heptaphyllum* (black breu)	Treatment of headaches (inhalation); treat pain and inflammation (plasters)		[25]
*P. heptaphyllum* (black breu)		δ-3-carene + *iso*-sylvestrene (79.5%)	[15]
*P. heptaphyllum* (black breu)		δ-3-carene + *iso*-sylvestrene (56.4%), *p*-cymene (14.0%), limonene + β-phellandrene (6.8%)	[15]
*P. heptaphyllum* (black breu)		*p*-cymene (33.0%), δ-3-carene + *iso*-sylvestrene (14.7%)	[15]
*P. heptaphyllum* (breuzinho)		δ-3-carene + *iso*-sylvestrene (69.0%), *p*-cymene (6.4%), limonene + β-phellandrene (5.7%)	[15]
*P. heptaphyllum* subsp. *heptaphyllum*		*p*-cymene (39.9%), *n*-tetradecane (13.4%), dihydro-4-carene (11.7%), α-phellandrene (7.4%)	[26]
*P. heptaphyllum* subsp. *ulei*		terpinolene (42.3%), *p*-cymen-8-ol (13.6%), limonene (11.9%)	[26]
*P. icicariba*		α-pinene (5.6–7.7%), *p*-cymene (20–40%), limonene (5.8–8.0%), α-terpinolene (5.8–31%), *p*-cymen-8-ol (10–26%)	[27]
*P. neglectum*	Traditional remedy for inflammations, as an inhalant to clear respiratory and bronchial passages, wound healing. Antibacterial, disk diffusion assay (*Bacillus subtilis*, *Staphylococcus aureus*)	Fresh resin: *p*-cymene (5.2%), durenol (15.6%), α-terpineol (6.9%), piperitenone (25.4%), thymol (17.5%), methyl eugenol (9.2%)	[28]
*P. occultum* (white breu)	burning and inhaling smoke to treat headache	*p*-cymene (10.4%), limonene + β-phellandrene (41.1%), α-terpineol (30.9%), α-pinene (8.0%)	[15]
*P.* cf. *opacum* (surucuba)		*p*-cymene (6.6%), α-*neo*-clovene (5.3%), α-*neo*-callitropsene (7.3%), γ-cadinene (14.4%)	[15]
*P. strumosum* (white breu)	burning and inhaling smoke to treat headahce	α-pinene (57.7%), β-pinene (9.3%), *p*-cymene (9.2%), limonene + β-phellandrene (10.8%)	[15]

^a^ Rüdiger and co-authors have reviewed the chemistry and pharmacology of *Protium* in 2007 [2]. This table includes analyses reported since 2007.

**Table 2:** Chemical composition of the oleoresin essential oil of *Protium amazonicum* from Ecuador.

RI^calc^	RI^lit^	Compound	%
779	780	Toluene	0.2
925	930	α-Thujene	0.7
932	939	α-Pinene	4.0
947	952	α-Fenchene	0.2
949	954	Camphene	0.1
970	972	3,7,7-Trimethyl-1,3,5-cycloheptatriene	1.4
972	975	Sabinene	0.1
977	979	β-Pinene	1.0
1000	1002	δ-2-Carene	0.1
1007	1002	α-Phellandrene	0.5
1010	1011	δ-3-Carene	47.9
1017	1017	α-Terpinene	0.4
1019	1026	*o*-Cymene	0.3
1024	1024	*p*-Cymene	4.1
1029	1029	Limonene	5.1
1030	1029	β-Phellandrene	0.4
1032	1031	1,8-Cineole	0.7
1057	1059	γ-Terpinene	0.5
1072	1072	Pinol	0.2
1080	1085	*m*-Cymenene	1.8
1085	1088	Terpinolene	0.7
1090	1091	*p*-Cymenene	3.2
1095	1099	α-Pinene oxide	0.1
1141	1139	*trans*-Pinocarveol	0.1
1142	---	2-Isobutylnorbornane	0.7
1147	1146	Camphor	0.3
1149	1147	*trans*-Dihydro-α-terpineol	0.5
1153	1150	Eucarvone	0.3
1162	1170	α-Phellandren-8-ol	1.9
1170	1160	*iso*-Borneol	0.3
1171	---	β-Phellandren-8-ol	0.9
1174	1169	Borneol	0.6
1180	1179	*m*-Cymen-8-ol	4.8
1183	---	*p*-Isobutyltoluene	0.3
1184	1182	*p*-Methylacetophenone	0.1
1186	1182	*p*-Cymen-8-ol	1.7
1188	---	(*Z*)-β-Ocimenol	0.2
1195	1188	α-Terpineol	5.5
1207	1205	Verbenone	0.2
1210	1217	4-Methyleneisophorone	3.0
1220	---	2-Carone	0.9
1240	1238	(*E*)-Ocimenone	0.2
1242	1241	Cuminal	0.1
1243	1243	Carvone	0.2
1246	1248	Car-3-en-2-one	0.4
1248	1247	Carvotanacetone	0.2
1253	1252	Piperitone	0.1
1264	1268	3,5-Dimethoxytoluene	0.2
1277	1275	Phellandranal	0.3
1290	1290	Thymol	0.2
1296	1299	Carvacrol	0.3
1419	1419	β-Caryophyllene	0.1
1433	1434	α-*trans*-Bergamotene	0.9
1581	1583	Caryophyllene oxide	0.2
		Total identified	99.6%

RI^calc^ = Retention indices calculated in reference to a homologous series of *n*-alkanes on a ZB-5MS column. RI^lit^ = Retention indices from the literature [30].

**Table 3:** Enantiomeric excess (ee) and distribution (ed) of monoterpenoids in the resin oil of *Protium amazonicum*.

Compounds	Relative %	ee (%)	ed [(+) to (−)] (%)
α-Thujene	0.7	45.6	27.2 to 72.8
α-Pinene	4.0	41.8	29.1 to 70.9
β-Pinene	1.0	45.6	27.2 to 72.8
δ-3-Carene	47.9	100	0 to 100
Limonene	5.1	68.0	84.0 to 16.0
α-Terpineol	5.5	79.6	89.8 to 10.2
